# Valorization of Bio-Residues from the Processing of Main Portuguese Fruit Crops: From Discarded Waste to Health Promoting Compounds

**DOI:** 10.3390/molecules26092624

**Published:** 2021-04-30

**Authors:** Liege A. Pascoalino, Filipa S. Reis, Miguel A. Prieto, João C. M. Barreira, Isabel C. F. R. Ferreira, Lillian Barros

**Affiliations:** 1 Centro de Investigação de Montanha (CIMO), Instituto Politécnico de Bragança, Campus de Santa Apolónia, 5300-253 Bragança, Portugal; liegeaguiar@gmail.com (L.A.P.); freis@ipb.pt (F.S.R.); iferreira@ipb.pt (I.C.F.R.F.); 2 Nutrition and Bromatology Group, Faculty of Food Science and Technology, University of Vigo, Ourense Campus, E32004 Ourense, Spain; michaelumangelum@gmail.com

**Keywords:** bio-residues, Portuguese fruit crops, bioactive compounds, apple, orange, pear

## Abstract

Food processing generates a large amount of bio-residues, which have become the focus of different studies aimed at valorizing this low-cost source of bioactive compounds. High fruit consumption is associated with beneficial health effects and, therefore, bio-waste and its constituents arouse therapeutic interest. The present work focuses on the main Portuguese fruit crops and revises (i) the chemical constituents of apple, orange, and pear pomace as potential sources of functional/bioactive compounds; (ii) the bioactive evidence and potential therapeutic use of bio-waste generated in the processing of the main Portuguese fruit crops; and (iii) potential applications in the food, nutraceutical, pharmaceutical, and cosmetics industries. The current evidence of the effect of these bio-residues as antioxidant, anti-inflammatory, and antimicrobial agents is also summarized. Conclusions of the revised data are that these bio-wastes hold great potential to be employed in specific nutritional and pharmaceutical applications.

## 1. Introduction

Recent statistics showed that European food processing units might generate approximately 100 Mt of waste and by-products each year, mostly comprising the production of drinks (26%), dairy and ice cream (21.3%), and fruit- and vegetable-derived products (14.8%) [1]. According to the Food and Agriculture Organization of the United Nations (FAO), one-third of the world’s food production (1.3 billion tons) is lost or wasted [2].

The term food loss is associated with food spoilage before reaching its final destination; in turn, food waste consists of food that is not consumed and is discarded or left to spoil by retailers or consumers. Despite being different concepts, food loss and food waste both cause a decrease in the availability of food for human consumption along the whole supply chain. However, food waste can still be suitable for human consumption. In this sense, the terms bio-waste and bio-products also arise. In the present review, the term bio-waste or bio-residue will be adopted, under different circumstances, to refer to waste, i.e., any product/compound without economic value generated from any process (e.g., apple pomace). On the other hand, the term by-product will be generally used to refer to products that are only discarded because they do not meet a specific production/consumption requirement. However, they maintain their physical-chemical or quality properties (e.g., a fruit that does not have the appropriate size). The FAO report showed that more than 40% of food losses in developed countries occur in retail and consumer markets [2,3].

Regarding fruits, there is a considerable percentage that reaches the consumer not as the whole fruit itself, but in processed formulations, such as juices or pulps. For example, tomatoes are frequently sold in the form of tomato paste, juice, or sauce. In some of these formulations, seeds, skin, and pomace must be separated, resulting in fruit bio-residues, commonly used in low-value applications such as feed or fodder [4,5]. As fruit bio-residues are considered food waste, their economic value is low. In this way, they may represent a financial problem for companies, especially those producing them in large quantities. In most cases, given the lack of applicability of these bio-residues, they do not present any economic advantage for the manufacturing units that need to dispose of them ecologically and responsibly. To reduce the potential environmental impact of these residues while providing additional economic benefits, the scientific community has been focused on this subject in the last years, seeking different valorization alternatives [3,4,6,7]. The food industry’s bio-residues have been identified as an excellent source of bioactive and functional compounds, with possible applications in nutraceutical and pharmaceutical formulations [6]. It is important to highlight that these residues can also be used to generate energy, either as heat, steam, or electricity, helping to reduce the energy invoice [7]. Biorefineries have stood out in this context, mainly concerning biomass conversion processes for the production of fuels, energy, heat, and value-added chemicals from biomass residues [8]. Furthermore, biorefineries have many environmental and economic advantages compared to traditional technology [9,10,11]. These aspects demonstrate the great importance and need for innovative research to discover suitable and under-valued agro-industrial bio-residues and by-products, as well as developing the most sustainable and efficient extraction methodologies to obtain bioactive compounds of interest [8].

This paradigm is also associated with currently relevant concepts, such as bioeconomy and circular economy [12], since the drastic increase in energy consumption and the deterioration of the environment forced us to retreat and move from a linear economy (dependent on fossil fuels) to a sustainable circular bioeconomy (based on green resources, energy, and methodologies, with zero-waste generation) [13,14]. Fundamentally, the intention is to replace the orthodox idea of the end of life with the concept of regeneration, increase the use of renewable energy sources, minimize the use of toxic chemicals and, in general, eliminate waste [8]. Given the world’s challenges related to climate change, resource depletion and energy, and food security, the circular economy is expected to develop sustainably [15].

It is a fact that the modern world has a severe problem with wasting food and by-products. Therefore, it is necessary to find sustainable solutions for these residues to be used at their full potential. Following this line of thought, this review’s main objective is to summarize the bioactive compounds present in the main Portuguese fruit crops and their biological activities, further evaluating their potential applications.

### The Current Status of Fruit Production in Portugal

**Figure 1:**
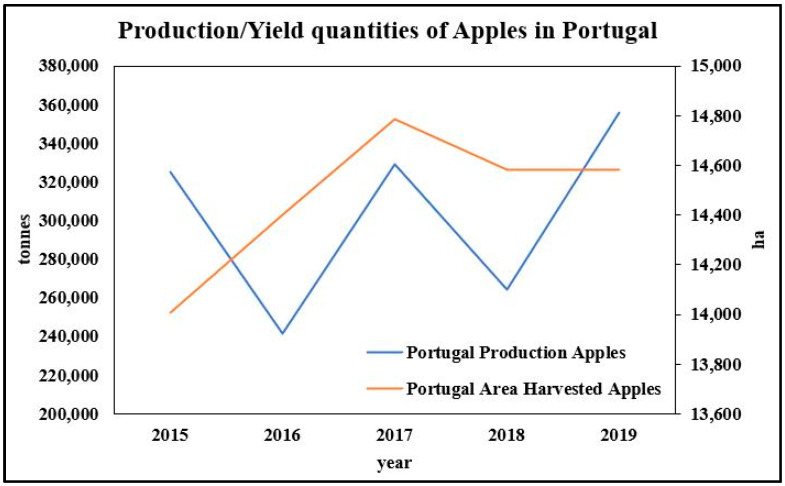
Production (in tons) and occupied area (ha) of apples in Portugal in the quinquennium 2015–2019. Source: Adapted from FAOSTAT (2020).

In Portugal, the estimated average production of apples is 265,000 t/year, being the main permanent crop in mainland Portugal, followed by orange and pear [16]. Currently, 14,580 ha are used for apple production in the country. Data indicate that in 2018 apple production was around 264,000 tonnes (Figure 1) [17]. Due to favorable weather conditions, in 2019 there was an increase in production, more significantly in the region of Trás-os-Montes (+65%) [18], reaching a national production of 355,700 tonnes.

Apple (*Malus* spp.) is one of the most popular fruits in the world. More than 95 countries have apple crops, mostly meant to meet the domestic needs of the population [19]. This high production may also be due to the fact that the beneficial effects of this fruit have been validated in the prevention of chronic heart and vascular diseases, respiratory and pulmonary disorders, diabetes, obesity, or cancer, among many others [20]. According to FAO statistics, apple is cultivated worldwide, and its global production exceeded 86 million tons in 2018, in an estimated area of 5 million hectares.

A significant part of apple production is processed and converted into juice or cider. The extraction of apple juice generates a solid residue, *apple pomace*, which is the main bio-residue obtained by crushing and pressing apples during the juice-making process and represents around 30% of the original fruits. This apple industry bio-residue consists basically of 94.1% of peels and 4.1% of seeds (data on a wet weight basis) [19,21,22]. Therefore, this industrial activity generates large quantities of underused bio-residues, which can be expensive and complex to remove. Thus, adding value to these materials might produce economic benefits, while reducing the huge volume of bio-residues demanding suitable disposal strategies [12].

**Figure 2:**
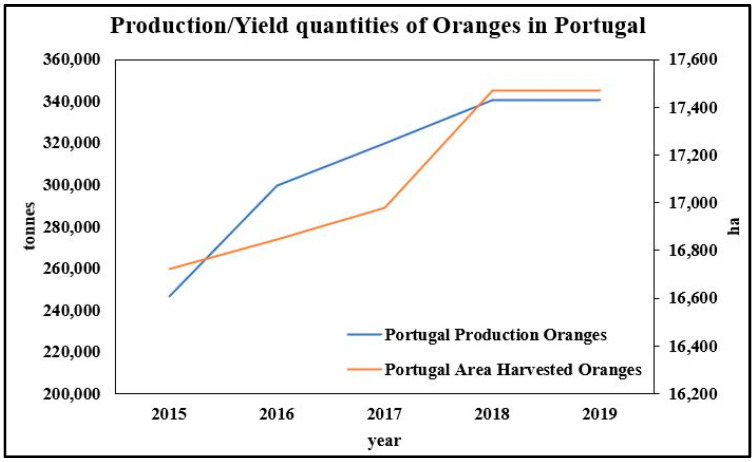
Production (in tons) and the occupied area (ha) of oranges in Portugal in the quinquenium 2015–2019. Source: Adapted from FAOSTAT (2020).

Orange (*Citrus sinensis* (L.) Osbeck) production in Portugal reached 340,000 t/year in 2018 and 2019, which corresponds to almost 18,000 ha in production area (Figure 2) [17]. In Portugal, citrus is mainly grown in the country’s southern region, namely in Algarve, where oranges are classified as a Protected Geographical Indication (PGI) fruit, granting a significant economic impact. As non-climacteric fruit, “Algarve Citrus” (“*Citrinos do Algarve*”) are harvested at their optimal ripe stage, when fruit internal quality attributes (IQA) comply with the requirements of the respective PGI normative reference [23,24]. During orange juice production, only about half the weight of fresh orange is transformed into juice. Meanwhile, the other half of the fruit’s weight generates large amounts of waste, including the peel, pulp, seeds, orange leaves, and whole orange fruits that do not meet the quality requirements [7,25]. It is important to highlight that these fruits are also known for their antioxidant properties that have beneficial effects on human health [26].

**Figure 3:**
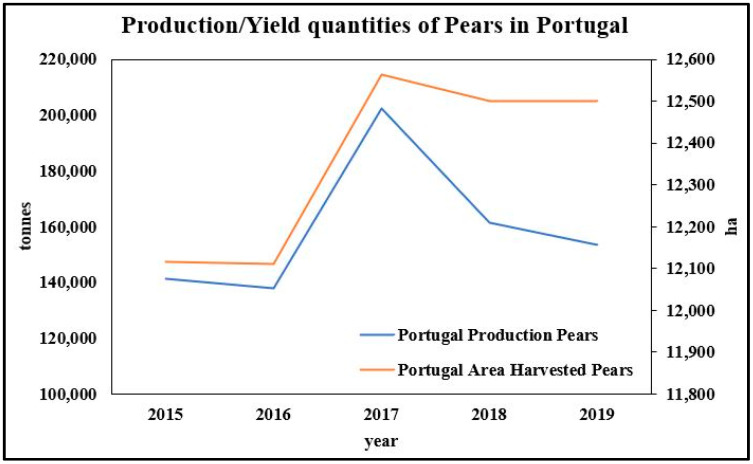
Production (in tons) and occupied area (ha) of pears in Portugal in the quinquenium 2015–2019. Source: Adapted from FAOSTAT (2020).

The production of pear (*Pyrus communis* L.) in Portugal in recent years has reached around 157,000 t/year, which corresponds to almost 12,500 ha in production area (Figure 3) [17]. It is the fifth most widely produced fruit in the world, being harvested mainly in China, Europe, and the United States. Pears are typically eaten fresh and also used to produce juice, puree, and jam [27,28,29]. The pear’s popularity among consumers is due to its desirable flavor, high digestibility, attractive color, soft pulp, sweet taste, and subtle floral aroma. Regarding their chemical composition and related bioactive properties, pears’ phenolic composition and antioxidant activity vary widely between different cultivars, which is also due to the stage of ripeness and storage conditions [30]. Studies have shown that a more varied and much higher phenolic content was found in the pear’s skin than in the fruit’s pulp. Additionally, a higher content of total soluble phenolics, together with high antioxidant activity, was observed in bark extracts from different pear cultivars [31]. Phenolic compounds are also associated with the nutritional quality of fruits, as they contribute (directly or indirectly) to modulate taste and aroma and to protect fruits from oxidative deterioration, besides interacting with proteins, carbohydrates, and minerals [30].

## 2. Bio-Residues from Main Portuguese Fruit Crops Processing as Sources of Bioactive/Functional Compounds

Fruits’ residues, such as peel, pulp, seeds, pomace, oil cake, etc., are readily available and make up about 30–50% of the total weight. The usefulness of food waste can be assessed by its composition and the cost of extracting valuable compounds. Residual products can consist of bioactive compounds such as polyphenols or glucans [32,33].

Apple pomace has been suggested as a source of bioactive molecules such as dietary fiber, protein, biopolymers, and natural antioxidants [19,22,34]. Solid waste is a rich and heterogeneous mixture composed mainly of apple peel, seeds, and pulp [21,34,35]. Most apple compounds remain in the pomace, including insoluble carbohydrates (cellulose, hemicellulose, pectin and lignin), simple sugars (glucose, fructose, and sucrose), as well as small amounts of acids, minerals, proteins, and vitamins, among others [19]. Moreover, apple pomace has also been reported as a good source of a range of phenolic constituents with antioxidant potential and human health beneficial effects [21,36]. Other constituents less studied in apples, especially in the cuticle wax layer, such as terpenoids, have been associated with antioxidant, antibacterial, and antitumor properties [19,34,35].

**Figure 4:**
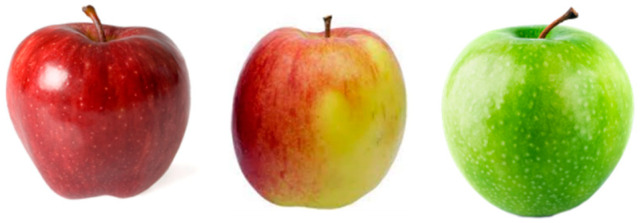
Color varieties of apples. Source: Adapted from [38] Cenário Hortifruti (https://saberhortifruti.com.br/maca/, accessed on 22 February 2021).

As is common knowledge, the color of the apples can vary between green, yellow, and red (Figure 4). These colors are related to the type of compounds present, namely chlorophylls, carotenoids, and anthocyanins. These type of compound compounds may therefore vary according to the cultivar [37].

Generally, apple pomace represents a low-cost source of phytochemicals and bioactive compounds, such as polyphenols, dietary fiber, pectin, triterpenoids, and volatiles [39].

**Figure 5:**
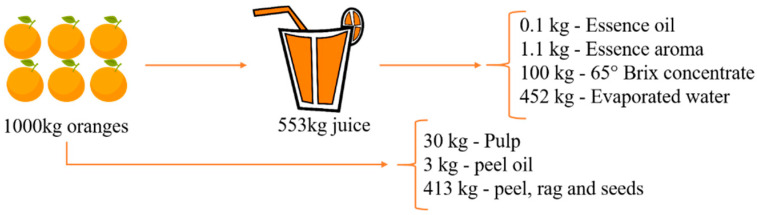
Products derived from whole oranges. Source: Adapted from [43].

Orange is a citrus fruit consumed in high quantities all over the world because it contains many nutrients including vitamin C, A, and B, minerals (calcium, phosphorus, potassium), dietary fiber, amino acids, and many phytochemicals, including flavonoids, triterpenes, phenolic acids, and carotenoids [7,40,41]. The valorization of residues requires knowledge of their chemical composition. Figure 5 shows the different types of products obtained from orange residues. Orange waste contains soluble sugars, cellulose, hemicelluloses, and pectin, its most important component [7]. The soluble sugars present in the orange peel are glucose, fructose, and sucrose. The insoluble polysaccharides of the orange peel’s cellular wall are composed of pectin, cellulose, and hemicelluloses. Pectin and hemicelluloses are rich in galacturonic acid, arabinose, and galactose, and also contain small amounts of xylose, rhamnose, and glucose [7,42].

**Figure 6:**
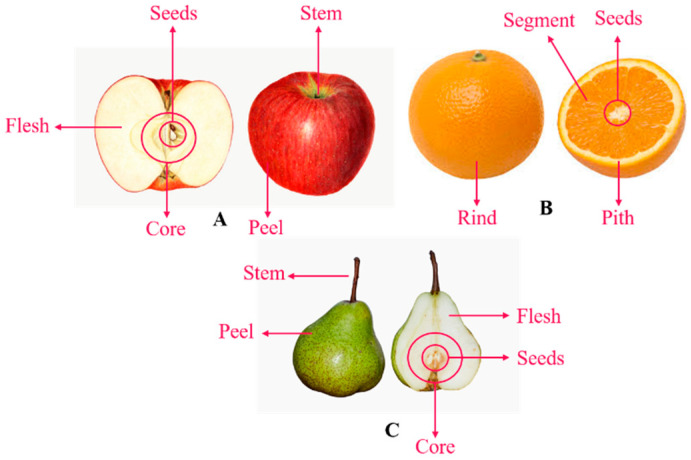
Scheme of each fruit cut in half representing the main components of (**A**) apple; (**B**) orange; (**C**) pear. Source: Adapted from [45,46].

Pear has high nutritional value with reasonable amounts of sugars, amino acids and minerals like sodium, potassium, calcium, magnesium and iron [44]. It also has a higher dietary fiber level than most common fruits and vegetables, giving excellent results in constipation and intestine inflammation [28]. Pears contain other nutritional and bioactive components as polyphenols [29]. There is little data available in the literature on pear pomace. Thus, it is assumed that the same bioactive compounds present in the fruit are present in its bio-residues. Figure 6 represents the main components of the three fruits covered.

The foremost biomolecules that may be found and contribute to bio-residues’ valorization are shown in Table 1.

As shown in Table 1, some biomolecules may be obtained from the residues of the industrial processing of the three fruits included in this review, sometimes being exclusive to just one of the three species. Below, the main bioactive compounds obtained from these residues are described.

### 2.1. Polyphenols–Biomolecules Common to the Three Fruit Crops

Polyphenols are an important group of secondary metabolites widely distributed in the plant kingdom [29]. Plants produce phenolic structures, mostly flavonoids and tannins, phenolic acids, lignans, and stilbenes as a defense mechanism against UV-light, parasites, other pathogens, and herbivores. Some of these molecules are pigments, attracting pollinators and thus contributing to plant seeds’ dispersion [3,13]. When consumed as part of the diet, these compounds are important to protect enzymes; provide vitamins, taste, and color; and avoid lipid peroxidation [55], having great utility as functional ingredients.

**Figure 7:**
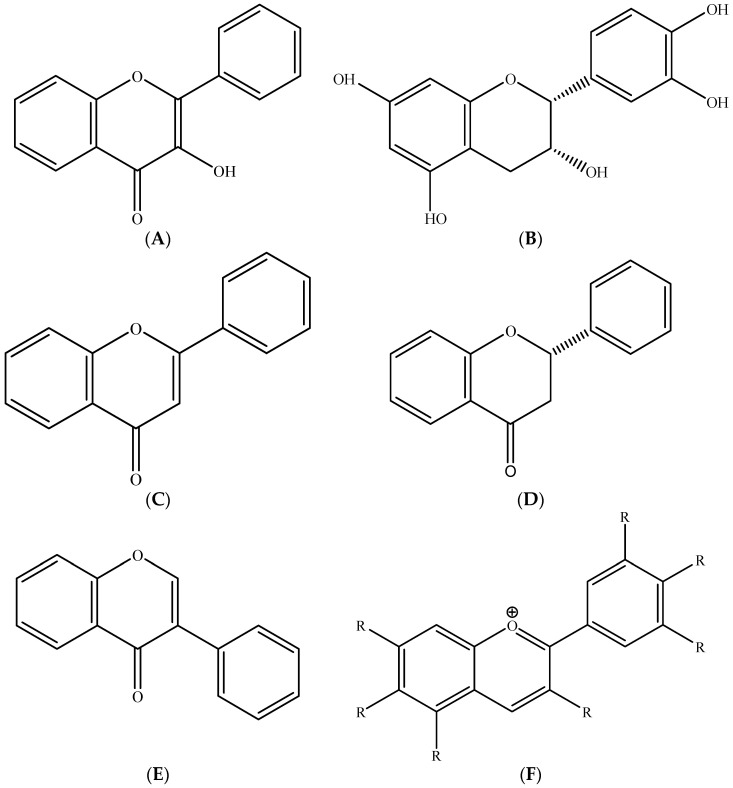
Subclass of flavonoids: (**A**) flavonols, (**B**) flavanols, (**C**) flavones, (**D**) flavanones, (**E**) isoflavones, and (**F**) glycosylated anthocyanidins where R = OH or R = CH_3_OH. Source: own authorship using ChemDraw Ultra software.

Flavonoids are among the most consumed polyphenols by humans. Given their wide presence in various fruits, vegetables, legumes, grains, and nuts, these compounds represent approximately two thirds of the phenols consumed, which is why the class is predominantly described. Flavonoids are polyphenols (phenolic compounds with more than one hydroxyl group linked with an aromatic ring) present in most plants. Currently, there are more than 8000 flavonoid structures identified. This subclass of phenolic compounds includes flavonols (Figure 7A), flavanols (Figure 7B), flavones (Figure 7C), flavanones (Figure 7D), isoflavones (Figure 7E), and anthocyanins (Figure 7F) [3,56].

Flavonoids are associated with many health benefits. They are potent natural antioxidants that can improve human health by preventing oxidative stress, (neuro)degenerative diseases, and cardiovascular diseases [57,58]. Phenolic compounds contribute also to the sensory quality of fruit (color, astringency, bitterness, and flavor) [29,59]. Generally, phenolic compounds are more concentrated in fruit skin than fruit pulp [60,61].

**Figure 8:**
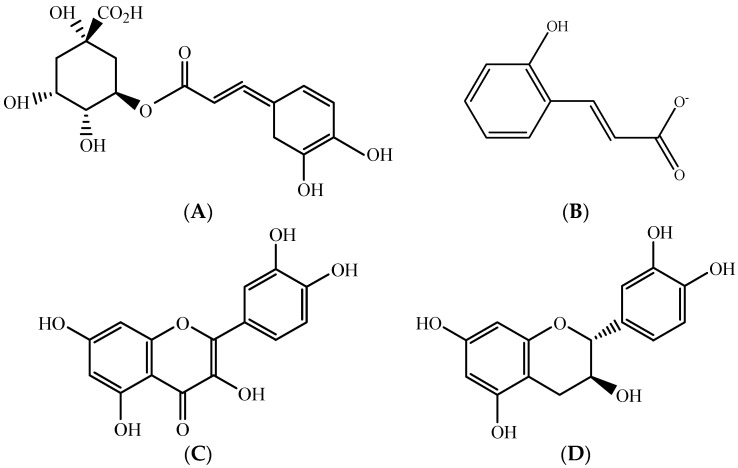
Chemical structure of main phenolic compounds in apple (**A**) chlorogenic acid, (**B**) hydroxycinnamate, (**C**) quercetin, and (**D**) catechin. Source: own authorship using ChemDraw Ultra software.

Apple pomace, which includes soft tissues, core, stems, seeds, and peels, is a good source of polyphenols such as chlorogenic acid (Figure 8A), hydroxycinnamates (Figure 8B), quercetin (Figure 8C), and catechin (Figure 8D) [13,62]. Studies show a wide variation in the polyphenolic content of different apple cultivars. The main compounds present in the polyphenolic fraction of apples were catechins and proanthocyanidins followed by hydroxycinnamates, flavonols, dihydrochalcones, and anthocyanins [63]. However, the number of individual compounds varies up to 30% from one year to another in the same cultivar [64].

**Figure 9:**
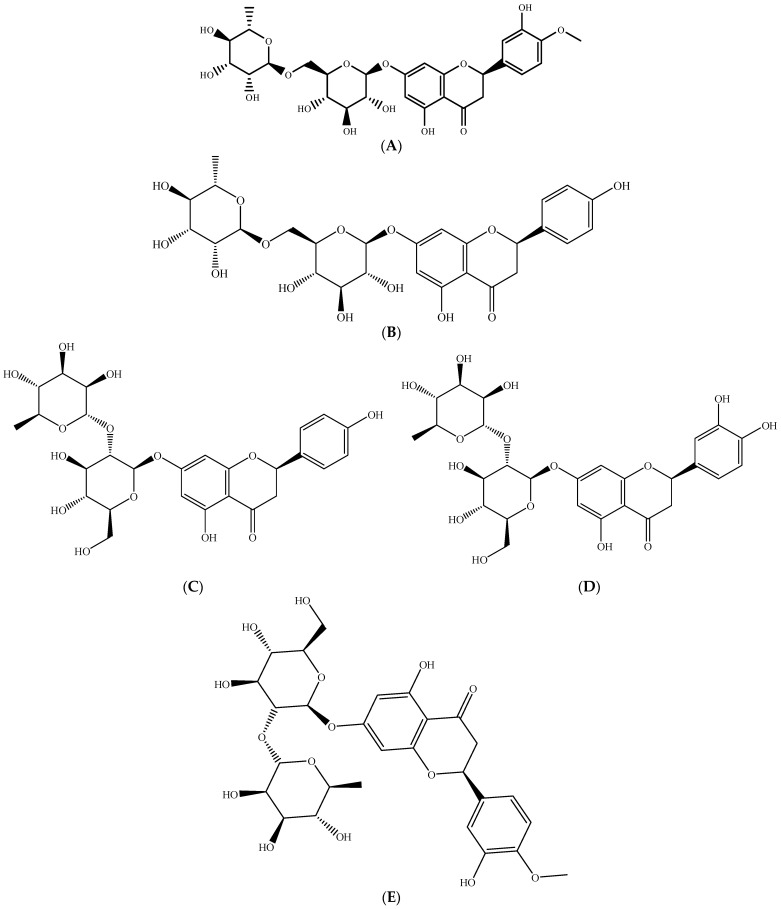
Chemical structure of main phenolic compounds in orange (**A**) hesperidin, (**B**) narirutin, (**C**) naringin, (**D**) neoeriocitrin, and (**E**) neohesperidin. Source: own authorship using ChemDraw Ultra software.

The profile of total flavanone of sweet and bitter oranges investigated by Farag et al. contained hesperidin (Figure 9A) and narirutin (Figure 9B) for sweet oranges and naringin (Figure 9C), neoeriocitrin (Figure 9D), and neohesperidine (Figure 9E) (hesperetin-7-neohesperidoside) for bitter oranges. A second class of citrus flavonoids, polymethoxy flavones (PMF), which are considered to be more biologically active than flavanones, although less abundant, have also been identified [49]. Fruit peels are richer in PMF than juice [65]. Likewise, it has been reported that citrus peel contains more polymethoxylated flavonoids than other parts of edible fruits [66]. Another study reported flavonoids as the most discriminating phytochemicals among citrus fruits, including orange, analyzed using hierarchical cluster analysis [67].

**Figure 10:**
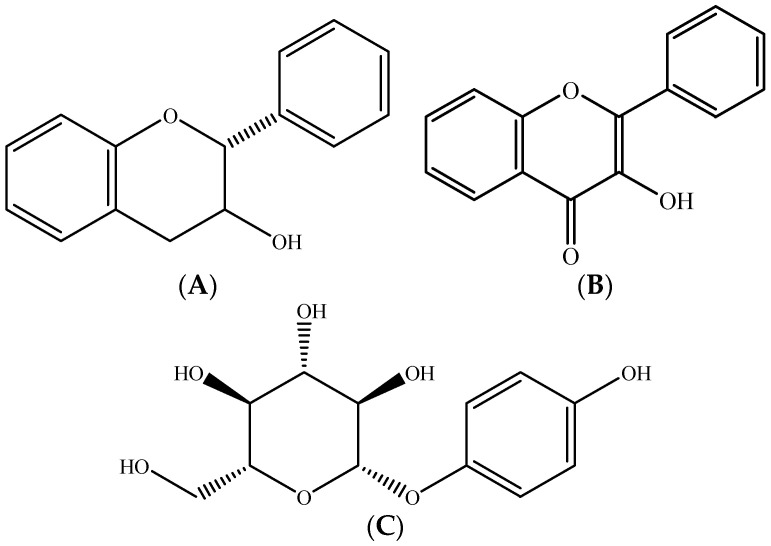
Chemical structure of main phenolic compounds in pear (**A**) flavan-3-ol, (**B**) flavonol, and (**C**) arbutin. Source: own authorship using ChemDraw Ultra software.

Pear polyphenols have diverse structures and belong to different classes, namely flavonoids (flavan-3-ols (Figure 10A) and flavonols (Figure 10B)), phenolic acids (hydroxycinnamic acids derived from caffeic acid and *p*-coumaric acid), and simple phenolics (glycosylated hydroquinone: arbutin (Figure 10C)) [61,68,69]. Many studies on polyphenol composition conclude that hydroxycinnamic acids and arbutin are the main phenolic compounds in pear [29,44,70,71,72].

### 2.2. Pectins–Biomolecules Common for Apple and Orange Crops

**Figure 11:**
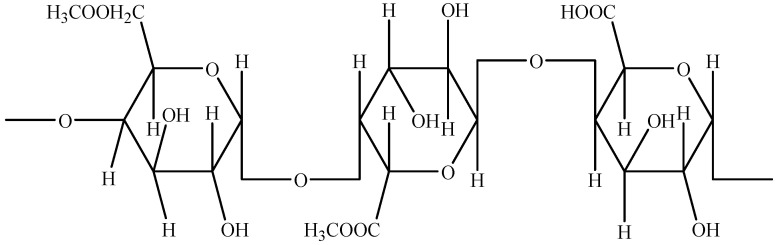
Chemical structure of pectin. Source: own authorship using ChemDraw Ultra software.

Pectin (Figure 11) is a structural heteropolysaccharide of D-galacturonic acid that can be organized into three different structures, namely homogalacturonane, ramnogalacturonane I, and ramnogalacturonane II [1,12]. Despite integrating soluble dietary fiber in several fruits and vegetables [73], the main sources of pectin at the industrial level are represented by apple pomace [74], citrus peel [7,75], and sugar beet pulp [76]. Dried apple pomace, citrus peel, and sugar beet treated with hot diluted mineral acid resulted in the production of 10–30% of pectins and flavanones [47]. The content of pectin (powder) in apple pomace, spent guava extract, and lemon peel in optimized conditions was found between 0.05 and 0.06% [77].

Apples contain approximately 2.21 g/100 g of total fiber. Of that, 70% is insoluble fiber, including cellulose and hemicellulose, and 30% is soluble fiber, mainly pectin [78]. Pectins are complex polysaccharides present in the cell wall of higher plants, which are not metabolized in the upper digestive tract in humans [79]. Beneficial health effects of pectin are attributed to its ability to lower cholesterol [80], slow down glucose absorption, and increase colonic short-chain fatty acid (SCFAs) production [78,81].

**Figure 12:**
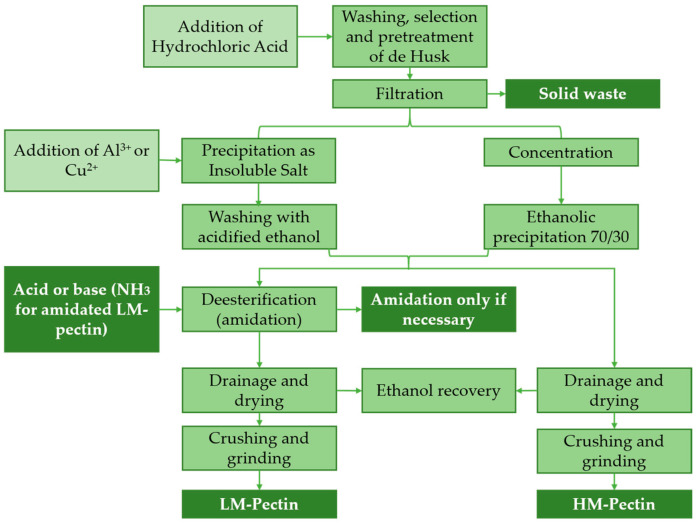
Manufacturing process of pectins started by acid-hydrolysis. LM-Pectin: low methoxyl pectin and HM-Pectin: high methoxyl pectin. Source: Adapted from [83].

Pectin can be obtained from pomace generated as a residue from the processing of citrus fruits, grapes, and apples with a wide range of uses in the pharmaceutical, cosmetic, and food industries as thickening and gelling agents to improve food texture [7,47,82]. For pectin extraction, two general processes are used: (1) those that separate pectins from most of the other materials by precipitation with alcohol and (2) those that precipitate pectins as an insoluble salt with suitable multivalent metal ions (Figure 12) [83].

Among the health-promoting properties, the immunomodulatory activity of polysaccharides has been specified [84]. More and more polysaccharides isolated from medicinal plants are reported for their potent immunomodulating activities [84,85]. Acidic heteropolysaccharides could mediate the activity of certain immunological factors. Important factors from innate immunity are the complement system, NK-cells, phagocytes (macrophages, neutrophils, and dendritic cells), and their metabolites (monokines) and mediator cells (mast cells, basophils, eosinophils, and thrombocytes), producing pro-inflammatory molecules [84]. Immunostimulating polysaccharides modulate the intestinal immune system, which has been known to direct not only defensive but also regulatory functions of both mucosal and systemic immune systems through lymphocyte migration depending on homing receptor pattern [86]. Furthermore, the proposed therapeutic perspective of herbal polysaccharides, activating the complement system and immune cells, is an expression of tumor-preventing, antitumor, and antimetastasis activities [87].

### 2.3. Triterpenoids–Biomolecules Common for Apple and Orange Crops

**Figure 13:**
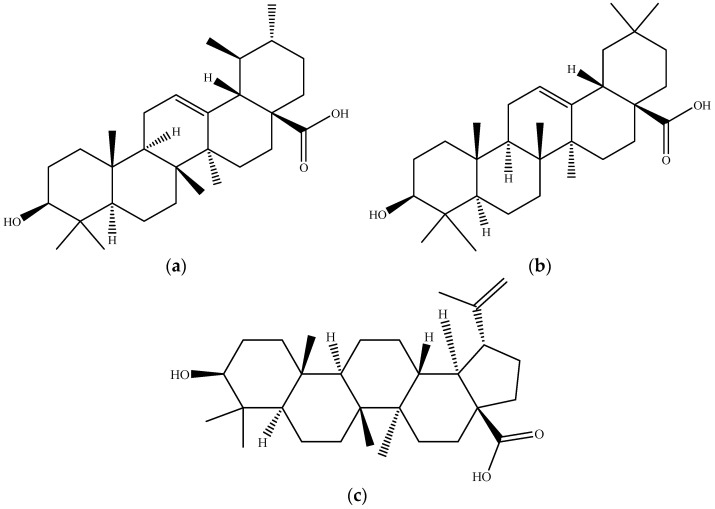
Chemical structure of (**a**) ursolic acid (UA); (**b**) oleanolic acid; and (**c**) betulinic acid. Source: own authorship using ChemDraw Ultra software.

Terpenoids are the most significant and widespread class of plant secondary metabolites synthesized from isopentenyl pyrophosphate oligomers [88], being composed of different isoprene units (C5H8) that may undergo chemical rearrangement [12,19,89,90]. The production of isoprene seems to be related to the mechanism that plants use to combat thermal and oxidative stress. Compounds derived from isoprene often undergo additional chemical modifications, such as oxidation or rearrangement of carbon structures. Triterpenoids are a group of compounds with 6 rearranged isoprene units (C30) with several beneficial biological activities and have, therefore, attracted research attention [19,89,91,92]. Regarding their application in food products, pentacyclic triterpenoids, particularly those with lupane, ursane, or oleanane structures, display several pharmacological activities, not to mention the fact of being devoid of prominent toxicity [19]. Among the ursan-based triterpenoids, ursolic acid (Figure 13a) is the main compound present in apple, followed by its oleanolic isomers (Figure 13b) and betulinic acids (Figure 13c) [12,89,92].

### 2.4. Carotenoids–Biomolecules Common for Apple and Orange Crops

Carotenoids are widespread secondary metabolites, biosynthesized by plants, algae, and some fungi and bacteria [93]. In addition to their relevance in terms of sensory quality, carotenoids are important from a nutritional point of view, being among the main precursors of vitamin A [94]. Moreover, there is a great deal of evidence associating high levels of carotenoid intake and a lower risk of developing chronic diseases such as heart disease [95], cancer, and macular degeneration [96], among other health benefits [97,98].

**Figure 14:**
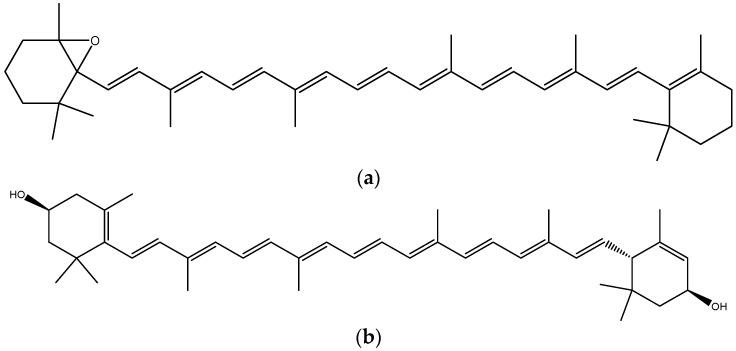
Chemical structure of (**a**) 5,6-epoxycarotenoid; (**b**) xanthophyll. Source: own authorship using ChemDraw Ultra software.

The color of orange varieties, one of the main attributes that affect customer acceptance, is mainly due to carotenoid pigments, of terpenoid origin (except for the shade of blood orange, which originates from anthocyanin pigments) [41,49]. The carotenoid profile of most sweet orange varieties is dominated by 5,6-epoxycarotenoid (Figure 14a) and xanthophyll (Figure 14b) [49,98].

Madrera et al. (2015) studied the characterization of aroma compounds from apple pomace and several uncommon terpenes were detected, all considered products of oxidative degradation of structural carotenoids (e.g., β-ionone, pseudoionone, β-dam-ascenone, 6-methylhept-5-en-2-one, 6-methylhept-5-en-2-ol, 6-methylhepta-3,5-dien-2-one, 2,6-dimethylhept-5-enal, 2,6-dimethylhept-5-en-1-ol, nerylacetone, and 6,10-dimethylundeca-5,9-dien-1-ol). Cyclic carotenes from apple, such as β-carotene, produce the cyclic terpenoids ionone, pseudoiononeor damascenone, while acyclic carotenes, such as lycopene, produce linear derivatives. As in the autoxidation of unsaturated fatty acids, oxidative activity occurs due to fruit damage during processing. Hence, these components are found in apple pomace both before and after fermentation [99].

### 2.5. Limonoids–Biomolecules Common for Orange Crops

**Figure 15:**
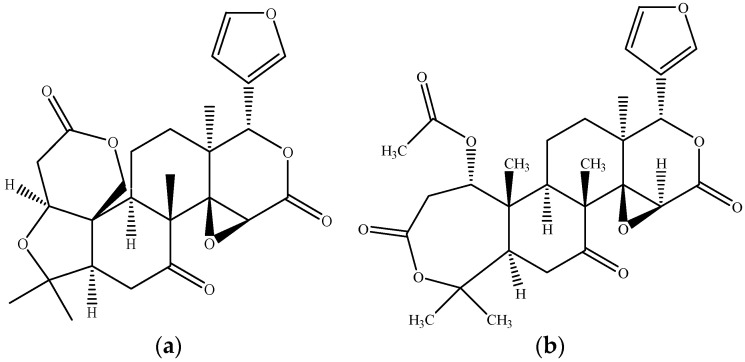
Chemical structure of (**a**) limonin; (**b**) nomilin. Source: own authorship using ChemDraw Ultra software.

Limonoids are a prominent group of oxygenated triterpenoids, present in the pulp, seed, and peel tissues of oranges [100]. Both lactones and glucosides are soluble in water but do not contribute much to citrus fruit taste. In contrast, limonoid aglycones, such as limonin (Figure 15a) and nomilin (Figure 15b), impart the sour taste of bitter orange [49].

## 3. Evidence-Based Bioactive and Therapeutic Potential of Bio-Residues Generated from Apple, Orange, and Pear Processing

As mentioned throughout this work, non-edible bio-residues from fruits can be equally (and often more) rich in compounds of interest as the edible parts. In this way, their extracts can be an interesting source of bioactive compounds that, in addition to bringing benefits to human health, can constitute possible alternatives to the wide use of artificial components in various products, such as food additives, among other applications.

The following section summarizes the health-promoting properties of specific compounds found in apple, orange, and pear bio-residues.

### 3.1. Antioxidant Activity

Regarding food, the definition of “antioxidant” is somewhat controversial. In the European legislation, antioxidants are defined as “substances which prolong the shelf-life of foods by protecting them against deterioration caused by oxidation, such as fat rancidity and color changes” [101]. On the other hand, according to the Institute of Medicine (US), “a dietary antioxidant is a substance in foods that significantly decreases the adverse effects of reactive species, such as reactive oxygen and nitrogen species, on normal physiological function in humans” [102]. In the food industry, antioxidants are used to extend food shelf-life by preventing the oxidation of lipids and vitamins in foodstuffs, and, consequently, rancidity or other undesirable flavors [3,103].

For example, rosmarinic acid has been approved as a natural antioxidant since it has antimicrobial, antiviral, antioxidant, and anti-inflammatory activities, making it a valuable component for the pharmaceutical, food, and cosmetic industries [104,105]. Other naturally active antioxidant agents, such as linalool [106], nisin [107], cinnamaldehyde [108], curcumin [109], red beet [110], ascorbic acid [111], tea polyphenols [112], ferulic acid [113], and quercetin [113], have been used as functional additives due to their antioxidant properties, including free radicals scavenging.

Antioxidants help to prevent several human diseases. Citrus seeds and peels have proved to be a good source of flavonoids such as eriocitrin, naringin, narirutin, and hesperidin, holding antioxidant properties. Peels are also a rich source of limonoids-nomilinic acid, nimolin, and limonin, which have been demonstrated to be effective antimicrobial, antiviral, and antibacterial compounds [13,114].

It is known that phenolic compounds (such as those present in apple pomace) can inhibit ROS; that is, they interrupt the cascade of free radical reactions in lipid peroxidation, in addition to other antioxidant effects depending on their structure [115]. Apple pomace, including seeds, is an excellent source of natural antioxidants, such as catechins, procyanidins, caffeic acid, phloridzin, phloretin glycosides, quercetin glycosides, and chlorogenic acid, among others. In recent times, due to the increasing interest in new natural sources of antioxidant compounds, apple pomace has been researched as a promising source of bioactive polyphenols [116,117,118,119,120]. Thus, the apple is an important resource with bioavailable polyphenols, such as flavonols, monomeric and oligomeric flavonols, dihydrochalcones, and anthocyanidins, among others [121]. The most abundant polyphenols that occur in apples are chlorogenic acid, floretine glycosides, and quercetin glycosides [122]. Specifically, the anthocyanin cyanidin 3-galactoside (Cy3-gal) showed strong antioxidant and cytoprotective activity [123]. The natural occurrence of Cy3-gal is limited to some fruit species such as *Pyrus communis* [72,124].

### 3.2. Anti-Inflammatory Activity

Inflammation is a biological response to various stimuli, including pathogens, harmful mechanical and chemical agents, and autoimmune reactions. It is the primary process by which the body repairs tissue damage and defends itself from stimuli, characterized by signs and symptoms, such as redness, swelling, heat, and pain [19,125,126]. Some classes of drugs (e.g., corticosteroids and non-steroidal anti-inflammatory drugs) used to treat inflammatory disorders can cause several adverse effects in addition to being expensive. Since antiquity, many people who suffer from inflammation have been treated with phytochemicals. This fact is evident from the discovery of acetylsalicylic acid. Thus, natural products offer great hope in the identification of bioactive compounds and their development in drugs for the treatment of inflammatory diseases [19,125]. Studies on the anti-inflammatory effects of various triterpenoids originating from natural products and chemical synthesis describe triterpenoids as powerful active agents acquired from natural products and excellent portions of synthesized compounds [19,39].

Inflammation underlies a wide variety of human diseases, and there is already evidence that polyphenols exert anti-inflammatory activities [78,127]. In a cross-sectional study of 8335 adults in the USA, apple intake was inversely associated with creative protein (CRP) levels, a biomarker of chronic inflammation [128]. Apple polyphenols, in particular procyanidins and florethine, demonstrate anti-inflammatory activities in vitro and can function as inhibitors of the expression of pro-inflammatory genes based on the transcription [129]. Several other human intervention studies have shown no association between apples [130], cloudy apple juice [130,131], or apple pomace [130] ingestion of inflammation markers.

Another important component of apples that may be partially responsible for its anti-inflammatory effect is fiber. In a meta-analysis of human intervention trials with increased consumption of dietary fiber, 6 out of 7 studies reported a significant decrease in CRP levels [132]. Whether the anti-inflammatory effects of apples are attributable to polyphenols, fiber, or synergistic interaction between them is yet to be confirmed [78].

The work by Dubois et al. [133] provided perhaps one of the first systematic studies demonstrating that citrus flavonoids, present in oranges, could in fact significantly inhibit inflammation in vivo. Several citrus flavanone glycosides, hesperidin and naringin, and their aglycones, administered at a dose of 45 mg Kg^−1^ day^−1^, were active against the inflammatory response of the rat granuloma pouch model [26,66].

The study of Huang et al. [134] suggested that the anti-inflammatory and antioxidant activities of pear extracts may be related to their phenolic contents. Many studies have also demonstrated that flavonoids and phenolic acids such as rutin, quercetin, luteolin, gallic acid, and caffeic acid produced significant antioxidant and anti-inflammatory activities [134,135,136].

### 3.3. Antimicrobial Activity

Antimicrobial resistance is one of the most severe worldwide concerns. Like antioxidant agents, antimicrobials can be synthetic or natural. The natural ones can be more efficient than or as efficient as the synthetic ones, having the advantage of being able to inhibit more than one microorganism at a time [137]. The use of new natural-based formulations to act as antimicrobial agents, particularly in synergistic combinations, also constitutes a particular promise to fight and overcome pathogens resistant to conventional antibiotic therapies [138].

In addition to their antioxidant activity, phenolic compounds might also be valuable for their antimicrobial properties, resulting mainly from their mechanism of action on cell membranes and their ability to bind to the lipid bilayer of the bacterial plasma membrane [3,139,140]. In fact, phenolic compounds integrate the plant defence system against microbial pathogens, such as fungi, bacteria, and viruses [141], making them potentially useful to fight the bacterial resistance problem [12,142,143,144]. Phenolic compounds can also inhibit the growth of microorganisms, such as *E. coli*, *K. pneumoniae*, *B. cereus*, *A. flavus*, and *A. parasiticus* [145]. Different concentrations of phenolic compounds exhibit different sensitivities towards different microorganisms [146,147]. Phenolic compounds are usually used as natural antioxidants in foods to extend the shelf life [148,149,150]. For example, phenolic compounds in cooked ground walnut showed high antioxidant activities during refrigerated storage [151]; phenolics in clove essential oil can enhance the stability of cake and other lipid foods for storage [152]; and a phenolic compound (rosmarinic acid) was used as a potential enhancer of functional properties in cupcakes [153].

Ursolic acid, present in apple pomace, is associated with antimicrobial effects against methicillin-resistant *S. aureus*, vancomycin-resistant *Enterococci*, *Streptococcus* sp., *Actinomyces* sp., and *Listeria monocytogenes* [39,154,155]. Synergistic effects of ursolic and oleanoic acid against *Mycobacterium tuberculosis* have been postulated [156].

Orange peel essential oil was reported to inhibit completely the growth of inoculated *S. aureus* in *Sardina pilchardus* from the second day till the end of the storage period with additional antioxidant activity required to suppress sardine rancidity. Such dual effects pose the oil to be used as a potent natural preservative with the ability of reducing lipid oxidation in sardines as well as protecting against the growth of common food-borne pathogens [49,157].

The study of Sato and Jin [158] succeeded for the first time in isolating the active substance that is essential for the antibacterial activity of an extract of succulent young shoot tissue of pear. The substance was identified as benzoquinone and the content in the aqueous extracts change with time, and changes parallel the changes in antibacterial activity.

### 3.4. Other Bioactivities

As explained in the current review, bio-residues and by-products which originated from the production line of fruit crops are particularly rich in bioactive compounds, which means that they have diverse biological activities and consequently a wide range of applications in several industry sectors. Therefore, in addition to its antioxidant, anti-inflammatory, or antimicrobial potential, other effects have also been reported in other available studies, which mostly refer to apple pomace [3].

Despite the existence of studies reporting a negative effect on serum total and HDL cholesterol level after apple pomace-based formulations intake [159], the most evident effect mediated by apple pomace based products consumption seems to be actually on cholesterol and triglyceride homeostasis [39]. These positive effects are mainly associated with the soluble dietary fiber content of the pomace [160,161]. The mechanism of action may be through the increase of cholesterol excretion and inhibition of gastrointestinal reabsorption of primary bile acids [162]. This promotes cholesterol uptake, synthesis, and turnover in the liver, leading to decreased cholesterol serum levels [39,163]. Growing evidence also indicates that the apple pomace polyphenols contribute to these positive effects [80]. The combination of apple pectin and a polyphenol-rich apple concentrate significantly decreased plasma cholesterol and triglyceride levels, as well as intestinal cholesterol absorption [39].

## 4. Potential Applications in the Food and Nutraceutical Industry

The bioactive compounds present in fruit bio-residues and by-products are extensively applied to food formulations to provide or enhance nutritional, sensory, and functional properties [164]. The application of these bioactive compounds can be direct, by applying the active compound to the food itself, or indirect, by applying these compounds, for example, in active food packaging [3]. The real-time quality evaluation of perishable products like fish, meat, fruits and vegetables, milk, and dairy products, is in great demand, particularly by developing intelligent packaging systems [165]. Thus, it is essential to innovate effective food quality indicators [32].

Bioactive compounds can be obtained in the form of extracts or essential oils. Several extraction processes can be applied, which are directly related to the chemical quality and the concentration of the antioxidant [166]. The extraction method’s choice must be carried out according to the extract/essential oil/active compound’s purpose, being phenolic compounds the mostly extracted compounds [3]. Several techniques can be used to extract bioactive compounds, such as supercritical extraction, hydrodistillation, distillation, or microwave-assisted extraction.

For the residues generated during the processing of oranges, the profitable alternative is the extraction of essential oils, that is, volatile oils extracted from the peel of citrus fruits [167]. Farhat et al. (2011) studied a new process for extracting essential oil from orange peel called microwave steam diffusion and concluded that it is an efficient method to extract essential oil from orange peel, providing good yields.

Orange essential oils have several applications in the pharmaceutical and food industries. D-limonene is an oily fraction and is considered one of the purest sources of monocyclic terpene [168]. Additionally, these oils may contain compounds with biological activity, such as limonoids and their glycosides, which proved to cause the inhibition of tumors induced in rats, mice, and hamsters [7,169]. The orange residue (solid and liquid) can be used to extract essential oils, while the solid residue, remaining after the extraction of the essential oil, can be reused to obtain cellulolytic fibers that are used as a support for semi-solid fermentation [170,171] or the production of dietary fiber [172,173]. The liquid residues remaining after the extraction of the essential oil are used in combination with the fibers as a substrate for the production of enzymes for semi-solid fermentation [7].

The production of essential oils from orange peel is economically viable since this bio-residue has high added value [174]. Citrus essential oils can be used directly as flavorings in foods and beverages and the production of medicines, cosmetics, and cleaning products [7,175]. Additionally, orange fiber was shown to be a promising fat replacer in ice cream production leading to a 70% reduction of fat without significantly affecting product attributes such as color, odor, and texture [176].

Owing to its nutritional value, apple pomace and pear pomace are prone to be used in manufacturing food products. In addition to nutritional components, apple and pear pomace contains high levels of fiber and polyphenols, which are known to play an important role in human health [177]. Some established examples, include the incorporation of finely ground apple pomace in several bakery products [177,178] or adding it to brown rice-based crackers [179].

## 5. Potential Applications in the Pharmaceutical and Cosmetic Industry

One of the most common treatments against skin infection or other dermal diseases is the use of broad-spectrum antibiotics. However, this may cause a negative influence on the natural microflora of the skin [180]. Therefore, the search for new drugs and new sources of antibacterial agents is of utmost importance [12,154].

A recent in vitro study validated the inhibitory capacity of apple phenols over the fat production in sebaceous cells [181]. This suggests that these phenolic compounds may be useful in the regulation of sebum production, alleviating skin diseases like acne (in which fat production is altered) [182]. Using natural-based products is becoming increasingly popular in the treatment of acne, inhibiting the associated inflammation symptoms [183].

Besides, several studies (in vitro and in vivo) have reported positive effects of phenolic compounds also against skin damages (e.g., wounds or burns), premature skin aging, psoriasis, rosacea, allergies, atopic dermatitis, and even cancer [184].

There are today numerous antioxidants in cosmetics that have the possibility of reducing the pigmentation level of the skin. Some of these substances possess a high tyrosinase-inhibiting effect that reduces total melanin production. Arbutin, a well-known natural antioxidant contained in bilberries and pears, has been developed as the strongest tyrosinase inhibitor and is widely utilized in cosmetics and personal-care products due especially to its mild side effects [185].

Humans with high carotenoid levels, substances present in oranges, have a younger skin appearance [186]. Beta-carotene, lycopene, lutein, and zeaxanthin are examples of carotenoids that play UV photoprotection [187]. As these molecules cannot be synthesized by humans, their ingestion is essential [188]. Beta-carotene is a precursor of vitamin A able to inhibit free radicals [189] and can accumulate in the skin, protecting it against UV-induced erythema, and thus, it is widely used as an oral supplement for sun protection [187,190].

## 6. Concluding Remarks and Future Trends

Apples, oranges, and pears are among the main fruit crops, being therefore responsible for producing a high number of bio-residues.

The bio-waste obtained from fruit processing has a high potential to be exploited as an innovative and competitive source of bioactive compounds, with potential applications in the food and nutraceutical industry and other sectors. These applications withstand a highly promising way of adding value to industrial bio-residues, for instance by reducing microbiological contamination and environmental impact, and minimizing costs to the manufacturing units. Despite extensive research on bio-waste, the discovery of new compounds is still expected, once preliminary studies point to promising antioxidant, anti-inflammatory, and antimicrobial activities. However, the deep characterization of these residues must be carried out to obtain safe and reproducible products. For example, future studies on the physiological effects of apple pomace should emphasize the material’s chemical characterization. This would facilitate the correlation of the biological activities with the pomace constituents and the material’s standardization when intended for therapeutic use. Simultaneously, different extraction methodologies must be compared to optimize the yield and quality of the bioactive or functional compounds present in the bio-residue/by-product. Moreover, despite the lack of toxicity, it should be considered that the crops are heavily sprayed with pesticides, requiring the residues to be subjected to complete toxicological analysis before being processed to extract valuable compounds.

Triterpenoids and phenolic compounds are the most common results in the literature and are becoming increasingly popular due to their bioactive properties, such as antioxidant, antimicrobial, anti-inflammatory, anticancer, and hepatoprotective.

Finally, this review presented a comprehensive overview of the valorization of the main Portuguese fruit crop bio residues derived from the fruit processing industry, which should be more encouraged for alleviating pollution hazards encountered with its poor waste disposal. In addition to contributing to the circular economy, the use of these residues implies the high availability of bioactive compounds, which is interesting for nutraceutical and cosmetic applications.

## Figures and Tables

**Table 1:** Diversity of biomolecules extracted from bio-residues.

Extractable Biomolecules	Substrate	Applications	Reference
Pectin	Citrus fruit peel, apple pomace, sunflower heads, sugar beet, and wastes from tropical fruits	Natural polymer for drug delivery, gel formation, water binder, mucoadhesive polymer, and gelling agents to improve food texture	[47]
Dietary fibres	Apple pomace, pear pomace, and orange pomace	Production of β-glucosidase from orange pomace, a key enzyme that can prevent the discoloration of fruit juices, and treatment of intestinal dysbiosis	[48,49]
Flavanones	Citrus fruit peels and residues after pressing segments and seeds	Phytomedicines, nutraceuticals, anticancer, and antimalarial	[29,47]
Essential oils (matricine, chamazulene, α-bisabolol)	Oranges and chamomile	Food, cosmetics, and pharmaceutical industries, treatment of aliments, including digestion, sleep disorders, wound healing, and skin infections	[50,51]
Anthocyanins	Grape skins like red apples	Cosmetic manufacturing, food processing, pharmaceutical industry, and solar cell development	[52]
Phenolic antioxidants	Apple, pear and oranges	Anti-radical, anti-aging, anti-cariogenic, hypocholesterolic agent, glycemia regulator, cosmetics (hair and skin care, anti-decay, anti-cellulite), and slimming products	[29,53,54]
